# Slowly activating voltage-gated potassium current potentiation by ML277 is a novel cardioprotective intervention

**DOI:** 10.1093/pnasnexus/pgad156

**Published:** 2023-05-10

**Authors:** Sean Brennan, Abrar I M Alnaimi, Lauren R McGuinness, Muhammad I M Abdelaziz, Robert A McKenzie, Sophie Draycott, Jacob Whitmore, Parveen Sharma, Richard D Rainbow

**Affiliations:** Department of Cardiovascular and Metabolic Medicine & Liverpool Centre for Cardiovascular Sciences, Institute of Life Course and Medical Sciences, University of Liverpool, L69 3GE, L7 8TX, UK; Department of Cardiovascular and Metabolic Medicine & Liverpool Centre for Cardiovascular Sciences, Institute of Life Course and Medical Sciences, University of Liverpool, L69 3GE, L7 8TX, UK; Department of Cardiovascular and Metabolic Medicine & Liverpool Centre for Cardiovascular Sciences, Institute of Life Course and Medical Sciences, University of Liverpool, L69 3GE, L7 8TX, UK; Department of Cardiovascular and Metabolic Medicine & Liverpool Centre for Cardiovascular Sciences, Institute of Life Course and Medical Sciences, University of Liverpool, L69 3GE, L7 8TX, UK; Department of Cardiovascular Sciences, College of Life Sciences, University of Leicester, LE1 7RH, L7 8TX, UK; Department of Cardiovascular Sciences, College of Life Sciences, University of Leicester, LE1 7RH, L7 8TX, UK; Department of Cardiovascular and Metabolic Medicine & Liverpool Centre for Cardiovascular Sciences, Institute of Life Course and Medical Sciences, University of Liverpool, L69 3GE, L7 8TX, UK; Department of Cardiovascular and Metabolic Medicine & Liverpool Centre for Cardiovascular Sciences, Institute of Life Course and Medical Sciences, University of Liverpool, L69 3GE, L7 8TX, UK; Department of Cardiovascular and Metabolic Medicine & Liverpool Centre for Cardiovascular Sciences, Institute of Life Course and Medical Sciences, University of Liverpool, L69 3GE, L7 8TX, UK

**Keywords:** slowly activating voltage-gated potassium current (IKs), cardioprotection, KCNQ1 (Kv7.1)/KCNE1, ischemic protection, myocardial infarction

## Abstract

Cardiovascular disease is thought to account for nearly a third of deaths worldwide, with ischemic heart disease, including acute coronary syndromes such as myocardial infarction, accounting for 1.7 million deaths per year. There is a clear need for interventions to impart cardioprotection against ischemia. Here, we show that the slowly activating voltage-gated potassium current (IKs) potentiator ML277 imparts cardioprotection against ischemia in cellular and whole-heart models by modulating the action potential duration. In three different metabolic inhibition and reperfusion models, an increased contractile recovery and cell survival was observed with ML277, indicative of protection. Finally, ML277 reduced infarct size in an ex vivo Langendorff coronary ligation model, including if only applied on reperfusion. In conclusion, potentiation of the IKs with ML277 imparted a cardioprotection that was equivalent to the protection reported previously by ischemic preconditioning. These data suggest that IKs potentiation may be therapeutically useful in acute coronary syndromes.

**Significance Statement**

The heart has an intrinsic ability to protect itself from some damage during acute coronary syndromes; however, activating this cardioprotection in man has proven difficult to translate to the clinic. Here, we show that this protection can be mimicked by the activation of one of the repolarization currents in the heart, slowly activating voltage-gated potassium current (IKs). Potentiation of this current may well represent a novel therapeutic target in reducing infarct size in acute coronary syndromes.

## Introduction

Cardioprotection is a term that can be applied to several scenarios that protect the myocardium from damage but is most commonly used in the context of ischemia. Since Murry et al.'s seminal 1986 publication demonstrating a protective effect in canine hearts imparted by a short period of ischemia prior to the main insult (1), the means of activating this protection clinically has been the subject of many investigations. In the nearly 40 years following this groundbreaking discovery, there have been a myriad of mechanisms hypothesized; however, the clinical translation of these has proven difficult.

Our, and others’, previous findings demonstrate that cardioprotection, imparted by several mechanisms that activate a PKC-dependent process, such as ischemic preconditioning (IPC) and adenosine and pharmacological interventions, causes modulation of the action potential and reduced consumption of ATP during ischemic conditions, leading to improved Ca^2+^ handling (2–7). From these findings, it was hypothesized action potential shortening, by selectively modulating an appropriate ion channel, would mimic the action potential shortening triggered by activation of the Reperfusion Injury Salvage Kinase (RISK) pathways, therefore reproducing the cardioprotective outcome pharmacologically.

Kv7.1 (KCNQ1) together with KCNE1 (previously referred to as MinK) form the complex that carries the slowly activating voltage-gated potassium current (IKs) in the heart are encoded by the *KCNQ1* and *KCNE1* genes, respectively. The stoichiometry of KCNQ1 and KCNE1 in the IKs complex has been heavily debated; however, reports have demonstrated mammalian cells contain two KCNE1 accessory subunits per channel complex (8). IKs is responsible for the slow component of the delayed rectifier K^+^ currents and plays a role in repolarization on the cardiac membrane potential following an action potential. The key role for IKs is underscored by various loss-of-function mutations causing long QT syndrome which decreased the current’s amplitude and consequently prolongs the cardiomyocyte APD (9).

ML277 is a potent Kv7.1 channel potentiator (10, 11) and selective against other Kv7 channels and other cardiac ion channels including hERG, Nav1.5, and Cav1.2 (12). ML277 preferentially activates Kv7.1 in the absence of the KCNE1 β-subunit and is less effective at potentiating KCNQ1 when saturated with KCNE1 (4:4 ratio). ML277 has been shown to potentiate native IKs in guinea pig and canine ventricular myocytes and human (iPSC)-derived cardiomyocytes (12, 13). The ability of ML277 to abolish calcium transient and action potential alternans in rabbit atrial myocytes (14), and reverse decreased IKs to partially restore action potential duration in LQT1 patient-derived human iPSC-cardiomyocytes (15), has led others to report IKs may have therapeutic value as an antiarrhythmic target.

Here, we demonstrate that ML277 can impart cardioprotection in cellular and whole-heart models of acute coronary syndromes, via a mechanism involving action potential shortening and reduced Ca^2+^ accumulation, similar to established cardioprotective pathways. These data suggest that IKs potentiation can confer cardioprotection and may be beneficial in acute coronary syndromes.

## Results

### Rat ventricular cardiomyocytes express an ML277-sensitive current

**Fig. 1.**
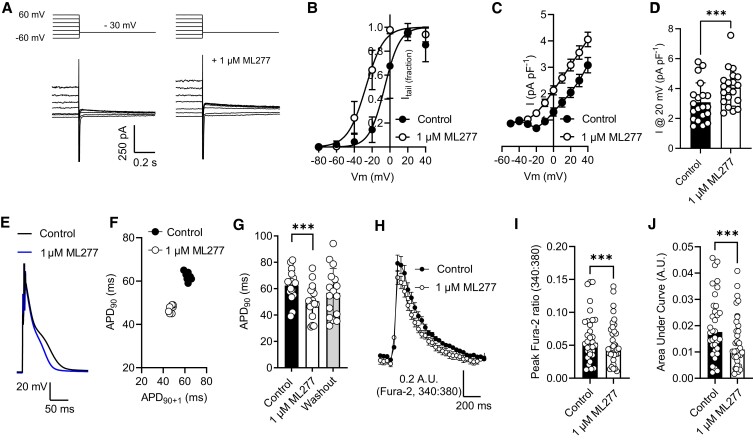
ML277 activates delayed rectifier K^+^ currents to shorten the cardiac action potential duration and reduce the Ca^2+^ transients. A) Example traces of currents recorded in a rat ventricular myocyte in control conditions and following 5 min of perfusion with 1 μm ML277 at the end of a 6 s depolarizing step, back to −30 mV to measure tail currents. B) Tail currents measured at −30 mV normalized to maximum tail current in the absence (control) and presence of 1 μm ML277 (*n* = 6 cells). Half maximal activation was −6 ± 2 mV in the absence and −29 ± 2 mV in the presence of ML277. C) Mean current–voltage relationship recorded from the end of a 6 s depolarizing step from a holding potential of −70 mV in the presence and absence of 1 μm ML277. D) Mean peak current amplitude in the absence (control) and presence of 1 μm ML277 (***P < 0.0001, paired *t*-test, *n* = 20 cells). E) Example traces of action potentials in the absence (control) and presence of 1 μm ML277. F) Poincare plot showing the variability of the action potential duration to 90% repolarized (APD_90_) in control and 1 μm ML277. G) Mean APD_90_ in the absence (control) and presence of 1 μm ML277 and washout in NT solution (***P < 0.0001, repeated measures ANOVA with Dunnett's posttest, *n* = 17 cells). H) Mean calcium transient traces from six cardiomyocytes recorded using Fura-2, in the absence (control) and presence of 1 μm ML277. I) Mean peak amplitude of calcium transients in the absence (control) and presence of 1 μm ML277 measured as a change in Fura-2 ratio. Peak amplitude calculated as the mean peak from 10 transients after 5 min of perfusion with control solution and 5 min of perfusion with 1 μm ML277 (***P = 0.0006, paired *t*-test, *n* = 34 cells). J) Mean area under the curve analysis for cells in I (***P < 0.0001, paired *t*-test, *n* = 34 cells).

To investigate whether treatment with ML277 was protective in isolated cardiomyocytes, an initial investigation was carried to confirm the presence of an ML277-sensitive current in rat cardiomyocytes. Voltage-gated K^+^ currents were activated using a whole-cell protocol, depolarizing from −60 to 60 mV in 20 mV steps for 6 s, followed by a step to −30 mV for 1 s. Cells were then perfused with Tyrode's solution containing 1 μm ML277 for 5 min, with the protocol repeated twice. Figure 1A shows the final 200 ms of the 6 s depolarization in the absence and presence of 1 μm ML277, showing a larger current in the ML277 trace in both the voltage steps and in the tail currents. As ML277 is far less effective on channels containing 4 KCNE1 subunits (12), these data imply native IKs channels in rat ventricular myocytes, as previously reported in canine and guinea pig (13), are not saturated with KCNE1. These data are similar to those recorded in HEK293 cells transiently transfected with KCNQ1/KCNE1 subunits (Fig. S1), where there was an increase in the whole-cell current. In both cardiomyocytes (Fig. 1A and B) and in HEK293 cells (Fig. S1D and E), there was a leftward shift in the activation curve (−6 ± 2 mV in the absence and −29 ± 2 mV in the presence of ML277), consistent with previous reports of the effects of ML277 on the IKs complex (12, 13). In both cardiomyocytes and HEK293 cells, there was a significant increase in the current recorded at 20 mV (Figs. 1D and S1C, respectively). Potentiation of a delayed rectifier current in rat ventricular myocytes would be expected to cause an action potential shortening by increasing the repolarizing K^+^ currents. To investigate this, action potential recordings were carried out using current-clamp recording. In these experiments, perfusion with 1 μm ML277 caused significant shortening of the action potential duration to 90% repolarized (APD_90_) (Fig. 1E–G), comparable with the shortening reported in rabbit atrial action potentials (14). Coupled to this, the calcium transients, as measured using the ratiometric indicator Fura-2, showed a reduced amplitude and reduced area under the curve in the presence of ML277 (Fig. 1H–J). For a more detailed analysis of the kinetics of the calcium transients, cells loaded with Fluo-4 were used and the transient duration, amplitude, and area under the curve were analyzed. These data showed a significant blunting of the transients, with an increased rate of return from peak to baseline (Fig. S2A–F). Contractile function of cardiomyocytes in the presence of 1 μm ML277 was also assessed using video-edge detection, demonstrating a significant reduction in contractile amplitude and area under the curve (Fig. S2G–L). Together, these data demonstrate that there is an ML277-sensitive current expressed in rat ventricular cardiomyocytes that is able to modulate the action potential duration and the calcium accumulation during each contractile cycle.

ML277 only potentiated the voltage-gated K^+^ channel component of the cardiomyocyte currents, having no significant effect on calcium or IK_1_ currents or hERG stably expressed in HEK293 cells (Figs. S3 and S4), analogous to previous reports of ML277 not altering other cardiac currents (12–14). Increasing ML277 concentration to 3 μm had no further potentiating effect on the shortening of the APD (Fig. S4), suggesting that ML277 has a limit to its effect on APD shortening. Conversely, the IKs channel blocker, JNJ303, inhibited IKs reversibly and did not affect other currents greater than the run down seen in these experiments (Fig. S5). To assess the potential translation of these data from rat to humans, the increase of IKs demonstrated in cardiomyocytes (Fig. 1) and in HEK 293 cells (Fig. S1) was simulated using the O’Hara-Rudy CiPA v1.0 (2017) model (16). In human, epicardial, myocardial, and endocardial models of IKs potentiation, achieved by shifting the activation curve leftwards by 30 mV, showed action potential shortening, an increase in IKs, and a decrease in the simulated intracellular calcium transient amplitude in all cell types modeled (Fig. S6).

### Treatment of cardiomyocytes with ML277 has a cardioprotective effect

Comparable with the effect of ML277 on action potential duration and calcium transients (Fig. 1), cardiomyocytes exposed to well-established cardioprotective stimuli also display a shorter cardiac action potential and reduced Ca^2+^ load during each contractile cycle (2, 17). These similarities led to the hypothesis that ML277 imparts cardioprotection against metabolic inhibition through shortening the action potential duration and improved calcium handling.

**Fig. 2.**
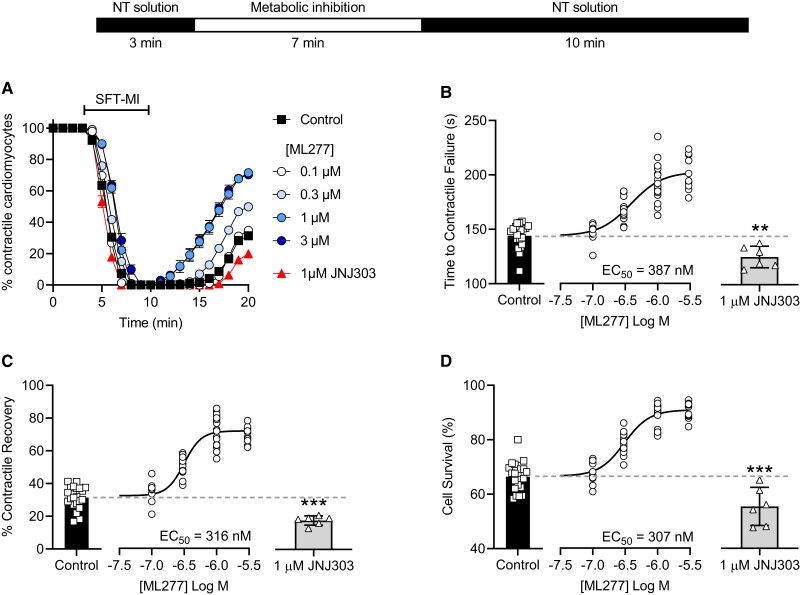
Perfusion of cardiomyoyctes with ML277 during a metabolic inhibition and washout protocol imparts cardioprotection. A) Time course of contractile cardiomyoyctes during the metabolic inhibition and washout protocol (outlined above) in control conditions, with 0.1–3 μm ML277, or with 1 μm JNJ303. B) Mean change in the time to contractile failure in the conditions outlined in A. The dotted line represents the mean value in control conditions [**P = 0.0029, unpaired *t*-test, JNJ303 (*n* = 6) vs control (*n* = 9)]. C) Mean change in the percentage of contractile recovery in the conditions outlined in A. The dotted line represents the mean value in control conditions [***P < 0.0001, unpaired *t*-test, JNJ303 (*n* = 6) vs control (*n* = 9)]. D) Data demonstrating the mean change in the percentage of cell survival in conditions outlined in A. The dotted line represents the mean value in control conditions [*P* = 0.0008, unpaired *t*-test, JNJ303 (*n* = 6) vs control (*n* = 9 (288 cells)]. For ML277 experiments, control (no drug) *n* = 21 (661 cells). For ML277, *n* = 12 (353), 14 (415), 18 (495), and 12 (408) experiments (cells) for 0.1, 0.3, 1, and 3 μm, respectively. Calculated EC_50_ values are included in each figure panel for clarity.

Cardiomyocytes were exposed to a metabolic inhibition and washout protocol that has been used in our (2, 17–20), and others’ (5, 21–27), previous publications to assess cardioprotection and toxicity. Figure 2A shows the mean time course of the percentage of contractile cardiomyocytes in control conditions, ML277 (0.1, 0.3, 1, or 3 μm), and with the selective IKs blocker JNJ303 (1 μm) (28). Consistent with established cardioprotective stimuli, ML277 caused a delay in the time to contractile failure (Fig. 2B), an increased contractile recovery (Fig. 2C), and an increase in cell survival (Fig. 2D). These effects were also replicated in an ischemic buffer as used previously in our group (2) and in a modified metabolic poison solution using sodium azide (Fig. S7A and B). Similar effects were observed in guinea pig cardiomyocytes, where both contractile recovery and cell survival increased in a concentration-dependent manner (Fig. S7C and D). Finally, JNJ303 demonstrated a cardiotoxic effect by causing a significant reduction in the time to contractile failure (Fig. 2B) and a decrease in the contractile recovery (Fig. 2C) and cell survival (Fig. 2D).

As further evidence of a cardioprotective effect of ML277 in isolated rat cardiomyocytes, the accumulation of intracellular Ca^2+^ (measured using Fluo-4) and mitochondrial depolarization (measured using tetramethylrhodamine methyl ester (TMRM)) were measured. The Fluo-4 fluorescence peak induced by metabolic inhibition was smaller in the presence of 1 μm ML277, and the initiation of this increase was delayed (Fig. S8A–C) (2). The mean time to mitochondrial depolarization was not different between the treated and nontreated groups; however, the peak amplitude of TMRM signal was significantly lower in the ML277-treated group (Fig. S6D–F). These data agree with our previous findings in cardioprotected cells (2). These findings demonstrate that ML277 has a protective effect on freshly isolated rat and guinea pig cardiomyocytes, as evidenced by improved contractile recovery, calcium handling, and cell survival plus reduced cell death and mitochondrial depolarization.

### ML277 is cardioprotective in a rat ex vivo whole heart coronary ligation model

**Fig. 3.**
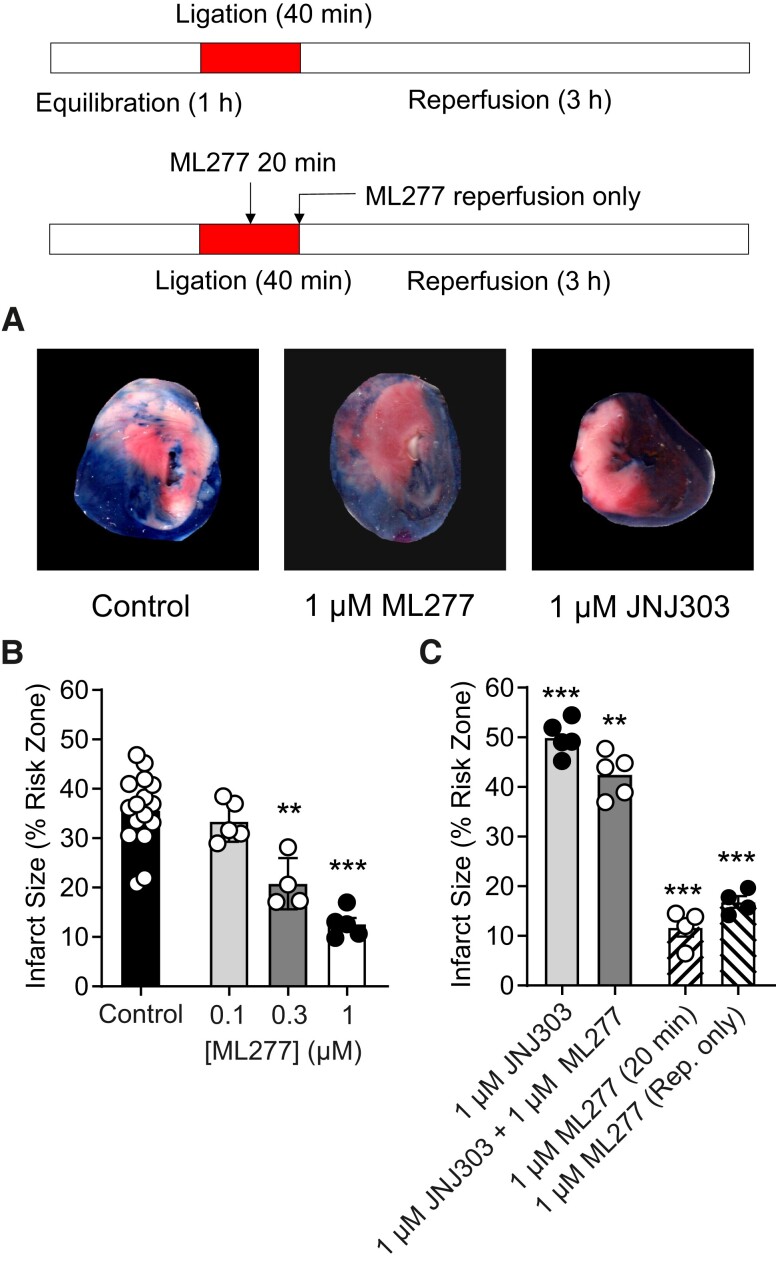
ML277 imparts cardioprotection to the whole heart in an ex vivo coronary ligation protocol. Whole hearts were perfused in a retrograde manner via the aorta for 1 h to acclimatize. The left LAD coronary artery was ligated for 40 min, followed by reperfusion for 3 h. A) Example images from heart slices stained with Evans blue indicator, showing areas unaffected by the coronary ligation, and living or infarcted tissue as shown using 2,3,5-triphenyltetrazolium chloride (TTC) staining (red and white staining, respectively). The percentage infarct size was calculated as the proportion of white staining as a fraction of the red and white stained regions using Image J. B) Bar chart showing the mean effects of 0.1–1 μm ML277 on infarct size when ML277 was included throughout the protocol (**P = 0.0002, ***P < 0.0001, one-way ANOVA with Dunnett's posttest, *n* = 16, 5, 4, and 5 hearts for control, 0.1, 0.3, and 1 μm ML277, respectively). The mean area at risk (red + white stained) was not significantly different between groups (one-way ANOVA). C) JNJ303 in the absence or presence of 1 μm ML277 significantly increased infarct size (***P < 0.0001, **P = 0.0031, one-way ANOVA with Dunnett's posttest, *n* = 16, 5, and 5 hearts for control, 1 μm JNJ303, and 1 μm JNJ303 with 1 μm ML277, respectively). ML277 introduced to the perfusing solution 20 min into the ligation or on reperfusion only significantly reduced the infarct size (***P < 0.0001, one-way ANOVA with Dunnett's posttest, *n* = 16, 4, and 4 hearts for control, application at 20 min, or on reperfusion, respectively. There was no significant difference between the area at risk (one-way ANOVA).

To assess whether ML277 can act as a cardioprotectant in the whole-heart, ex vivo left anterior descending (LAD) coronary artery occlusion was performed on a Langendorff system. ML277 reduced infarct size at concentrations >300 nm (Fig. 3A and B), consistent with the cellular metabolic inhibition data (Figs. 2, S5, and S6). Furthermore, IKs blocker JNJ303 increased infarct size, consistent with the cellular metabolic inhibition data (Fig. 3). Clinically useful therapeutics would most likely be administered either once ischemia was established or upon reperfusion. To model the clinical scenario, ML277 was applied either 20 min into the coronary ligation or on reperfusion only. In these hearts, the infarct size was significantly reduced compared with control conditions (Fig. 3C). These data show that ML277 imparted cardioprotection against ischemia and reperfusion injury to the whole heart.

## Discussion

**Fig. 4.**
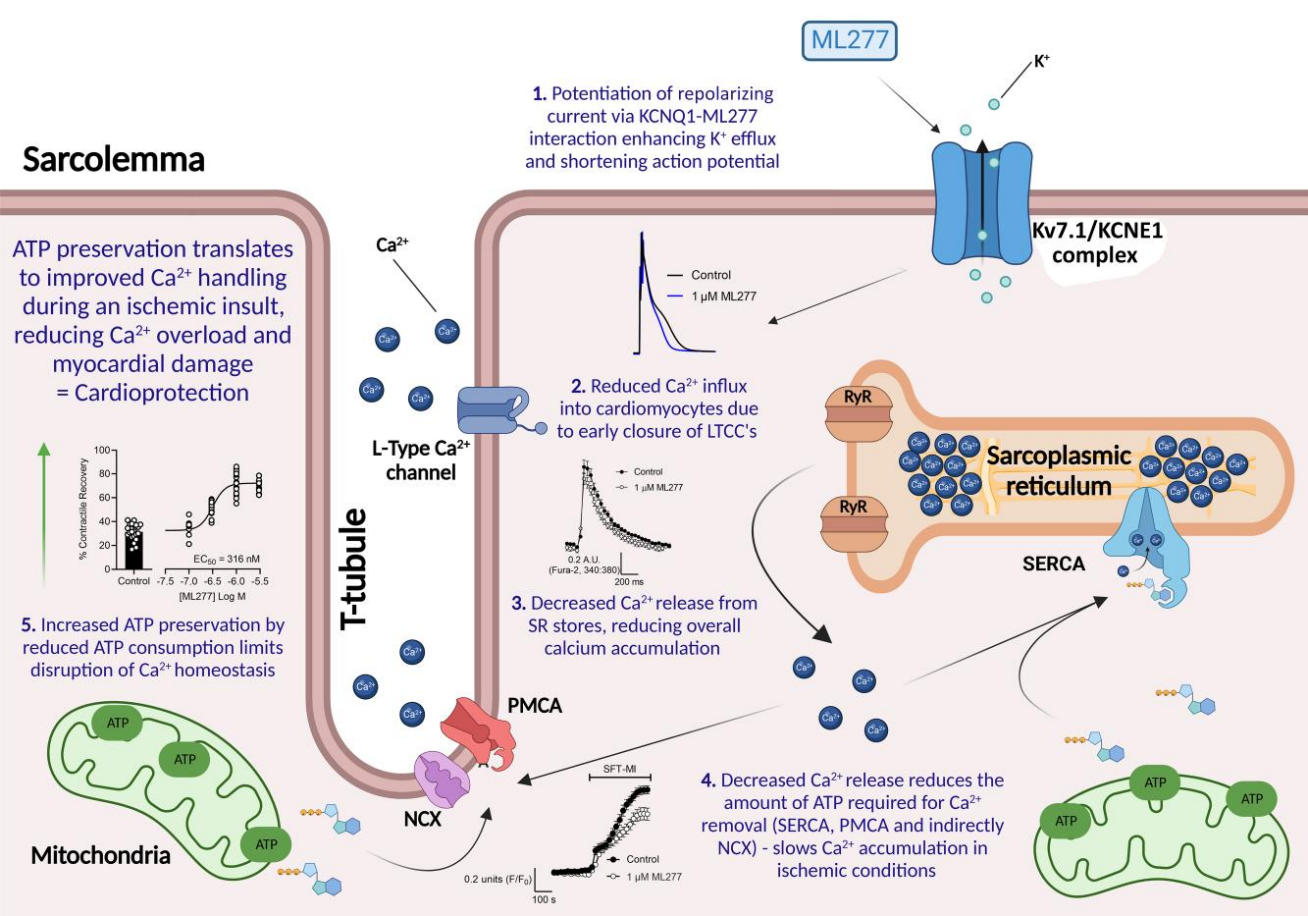
Cartoon illustrating the mechanism by which IKs potentiation by ML277 imparts cardioprotection. Graphic created with BioRender.com.

In this study, we demonstrate that the Kv71 activating compound, ML277, has a protective effect on cardiomyocytes and can reduce infarct size in a whole heart ex vivo model using coronary ligation. Our data show the activation of a delayed rectifier current in rat ventricular myocytes increases the current in the repolarizing phase of the rat ventricular action potential causing a significant APD_90_ shortening. The intracellular Ca^2+^ transients recorded from isolated cardiomyocytes show a reduced amplitude and area under the curve, suggesting reduced Ca^2+^ accumulation during each contractile cycle, thus reducing the ATP required to restore diastolic Ca^2+^ levels (Fig. 4). This reduced ATP consumption allows cells to maintain Ca^2+^ homeostasis for longer during metabolic inhibition and survive reperfusion more readily. We have previously demonstrated such time dependence to the reperfusion using this metabolic poison with ischemic preconditioned cells or treatment with adenosine, diazoxide, and pinacidil—all well-established cardioprotective interventions (2). Modeling the effects of IKs potentiation with the O'Hara-Rudy CiPA v1.0 (2017) model also showed that a marked shortening of the human APD corresponded with a reduced Ca^2+^ transient amplitude.

The use of direct ion channel modulation to impart cardioprotection is not unique. Pinacidil, among other ATP-sensitive potassium (K_ATP_) channel modulators, has been shown to have a cardioprotective effect. The activation of K_ATP_ channels is thought to be protective by limiting cellular excitability and so reducing ATP consumption; however, care should be taken with this as the known cardiac K_ATP_ complex, Kir6.2/SUR2A, is an abundantly expressed protein in the heart and its activation can cause significant cardiac action potential shortening and subsequent complete failure. Gain-of-function mutations of the cardiac K_ATP_ channel complex has been shown to cause short-QT syndrome and is proarrhythmic.

The established cardioprotection imparted by K_ATP_ channel activation demonstrates that direct increase in outward K^+^ efflux and subsequent action potential shortening can be cardioprotective; thus, it is plausible that IKs potentiation can be cardioprotective. Further support of the protective effect for IKs can be found in the literature where the contribution of IKs to repolarization during ischemia is increased, and in these conditions, IKs blockade with L-768,673 causes an increase in ventricular arrhythmias (29). This reported antiarrhythmic effect of IKs potentiation with ML277 is also a potential important therapeutic area where increasing the rate of repolarization, as our data suggests is occurring, could reduce the potential for early and delayed after-depolarizations triggered by high intracellular Ca^2+^ accumulations. A further consideration is the potential use of IKs as a drug target in certain long-QT syndromes, such as LQT-1, where the presence of some functional IKs could be potentiated to limit the incidence of fatal ventricular arrhythmias.

In contrast to these findings, it has reported that pan-Kv7 blockers improve infarct size in isolated rat hearts against ischemia reperfusion injury (30), but at an estimated concentration of 1 μm, the pan-Kv7 blocker XE991 did not improve infarct size during in vivo experiments (31). Although 1 μm XE991 does block Kv7.1 channels (32), Kv7.1/KCNE1 channels in oocytes display negligible block with 1 μm XE991 and 40% block to 10 μm after a 500 ms depolarizing step (33). It is, therefore, unlikely that the native cardiac IKs complex is susceptible to XE991 block over the duration of the cardiac action potential (<400 ms). Kv4.3, which contributes to the I_To_ current in myocytes (34), is blocked by XE991 with a reported IC50 of 43 ± 7 μm (32). This may explain the reported protective effect of 10 μm XE991 with in vitro experiments showing that 10 μm XE991 did not alter the rat atrial action potential duration and caused an ∼10 ms prolongation in guinea pig (31). Interestingly, the IKs blocker chromanol 293B appears to increase infarct size by >10%, but a larger n number was likely required to achieve significance (30).

The use of small molecules to modulate an ion channel activity can raise questions regarding selectivity; however, as previously reported, no major unspecific action of ML277 in the heart has been described (14). ML277 has been shown to be highly selective against other recombinant cardiac channels hERG, Na1.5, and Cav1.2 and other members of the Kv7 ion channel family (12). In a binding assay panel of 68 GPCRs, ion channels, and transporters, 10 μm ML277 was found to bind with only 6 assays conducted (35). The report states functional selectivity may be significantly better than suggested by binding activities and such discrepancies are not uncommon. For example, 10 μm ML277 was shown to have 80% inhibition against the hERG channel in a binding assay, but 30 μm ML277 had no activity against hERG in a functional assay. Our own whole-cell patch clamp data also demonstrate ML277 does not functionally inhibit hERG at 3 μm. In our assays, ML277 displayed cardioprotection at ∼300 nm which is close to the drugs reported EC_50_ for IKs. Furthermore, the selective IKs blocker JNJ303, which has no potent effects on the other cardiac ion channels (I_Na_, I_Ca_, I_Kr_, I_To_, and I_K1_) (36), displayed the opposite effect to ML227 and blocked the protection imparted by ML277. Although off-target effects can never be entirely ruled out, taking into account the known selectivity profile of the drugs used in this study, we conclude the effects observed are due to modulation of IKs.

Gain-of-function mutations of the IKs complex in the heart are rare and do give rise to short-QT syndromes in those affected. Such mutations tend to change the current’s properties from a slowly activating to rapidly activating, often by reducing its association with the KCNE1 subunit (37, 38). In comparison, ML277 causes a leftward shift in the activation curve for IKs as recorded in both ventricular myocytes and the cloned human subunits expressed in HEK293 cells. The current maintains its slowly activating component and does not excessively shorten the APD_90_, as would be seen in short-QT syndromes. This gives ML277 an advantage over sulfonylurea compounds that activate the K_ATP_ complex as cardioprotective agonists. The cardiac K_ATP_ complex has a Kir6.2 pore that forms a weakly rectifying K^+^ channel. This weak rectification property means that once activated, this complex passes K^+^ flux at any membrane potential with an increasing hyperpolarizing influence the more depolarized, or further from E_K_, the membrane potential becomes. It is entirely plausible that, with significant K_ATP_ complex activation, contractile failure could arise, which does indeed happen during significant periods of ischemia. Pharmacological potentiation of the IKs complex is unlikely to be so damaging to contractile function. Firstly, the effects of IKs on the cardiac action potential at rest are limited. Indeed, long-QT syndrome type-I (KCNQ1 loss-of-function mutations) is often subclinical and is only detected after a cardiac event or through genetic screening. The IKs complex, forming part of the delayed rectifier component of currents, is not a dominant repolarizing current until significant sympathetic drive. Additionally, this current is already active in cardiac cells, with ML277 potentiating that current. Opening of the K_ATP_ complex, however, is a pathophysiological event, usually associated with significant metabolic stress. Finally, the IKs complex carries a voltage-dependent current that is only activated on depolarization; therefore, potentiating this current will speed up repolarization and so is unlikely to prevent depolarization, in contrast to the Kir6.2/SUR2A K_ATP_ current. Our data, Supplementary Fig. S4, shows that increasing ML277 to 3 μm did not cause an increase in the effectiveness of the ADP shortening. These data suggest a “ceiling” to the effect that IKs potentiation has on APD in the context of pharmacological activation. This is potentially due to the slow kinetics of the IKs, where even with potentiation by ML277, the current still develops too slowly to cause a shortening that would be proarrhythmic. This would be an important consideration from a safety aspect where excessive shortening would not only be proarrhythmic, but also could significantly impair cardiac output to the detriment of the patient.

The mechanisms by which cardioprotection is imparted are complex and have been referred to as RISK pathways, which often involves the short-term activation of kinases (39–42). It has been suggested to comprise two parallel pathways, PI3K-Akt and MEK1-ERK1/2, being activated (42). Many of these potential pathways converge on PKC activation, particularly PKCε and PKCδ in the early and second windows of protection, respectively (27, 43–45). The activators of this pathway can be varied and can occur via metabolites released in stress conditions (e.g. adenosine (2, 46, 47)) or from endogenous receptor agonists (e.g. bradykinin and opioids (48–50)) but often occur in during a hypoxic state. In metabolically compromised tissue, these pathways may be difficult to modulate given the potentially low ATP availability for phosphorylation events which could render the pathway nonfunctional. Similarly, it is plausible that ATP and PIP_2_ depletion experienced during ischemia may underlie the reported downregulation of IKs, contributing to the proarrhythmic mechanism associated with poor outcomes from myocardial infarction (29). The administration of ML277, or an alternative IKs potentiator, could overcome this loss of expression so reducing proarrhythmicity, in addition to protecting against ischemic injury. We suggest IKs potentiation could therefore bypass the metabolic compromise or stress responses seen during ischemia, which may result in better clinical translation of a cardioprotective intervention.

In conclusion, we have presented data to demonstrate that ML277 has the potential to be a cardioprotective agonist that acts directly on a channel complex to reduce Ca^2+^ loading during ischemia and therefore preserve ATP for longer. This provides a mechanism by which the cell can delay, or at least slow, the Ca^2+^ accumulation that occurs with ATP depletion and therefore survive an ischemic insult more readily. The fact that this method of cardioprotection does not rely on the complex intracellular signaling via the RISK pathway is potentially of benefit given that few cardioprotective interventions with positive preclinical data translate to the clinic.

This work demonstrates that in ex vivo cellular and whole-heart models of ischemia reperfusion IK_S_ potentiation is beneficial. Further work is required to determine if this mechanism is suitable in higher species and whether similar effects can be observed in vivo.

## Materials and methods

### Cardiomyocyte isolation

The care and sacrifice of the animals conformed to the requirements of the UK Animals (Scientific Procedures) Act 1986 (2012 amendment). Ethical approval for all experimental procedures was granted by the University of Liverpool/Leicester's Animal Welfare and Ethical Review Body (AWERB_2018_44). Adult male Wistar rats (200–300 g) were killed by schedule 1 procedure of concussion and cervical dislocation. Cell isolations were carried out as previously reported (2, 17, 20). IPC was carried out on the whole whole-heart on a Langendorff canula using a stop–start perfusion protocol (2, 17, 20). (See supplementary methods section for full details.)

### Electrophysiology

Patch clamp recordings were carried out using isolated cardiomyocytes in the whole-cell configuration as described previously (2, 17, 20). (See supplementary methods for full details.)

### Metabolic inhibition

A well-established metabolic inhibition protocol was used to monitor cardiomyocyte contractility and survival. Both 2 mm cyanide and 1 mm iodoacetic acid were added to a substrate-free metabolic inhibition Tyrode's solution (SFT-MI) (see supplementary information for solution components) and perfused on isolated cardiomyocytes, simulated to contract via 1 Hz stimulation as described previously (2, 17, 20). (See supplementary methods for full details.)

### Fluorescence imaging

To record calcium transients, cardiomyocytes were loaded with 5 μm Fura-2-AM for 20 min at room temperature. Cardiomyocytes were stimulated to contract at 1 Hz using electric field stimulation (EFS) and perfused at 32°C. Data was acquired using WinFluor 4.1.6 software (Strathclyde University), with 340 and 380 nm excitation illumination provided by a PTI DeltaRam X monochromator. Emissions were collected using an Andor Zyla 4.5 camera at wavelength >520 nm. Images were acquired at a rate of 26 ratios per s.

### Ex vivo coronary ligation

A standard LAD coronary artery ligation model was used to cause an infarct to a Langendorff perfused heart. The artery was ligated for 40 min, followed by 3 h of reperfusion, where infarct was measured using tetrazolium chloride staining, as previously reported (17). (See supplementary methods for full details.)

## Supplementary Material

pgad156_Supplementary_Data

## Data Availability

The data that support the findings of this study are available in this manuscript and the Supplementary material.
