# Supplementary material for: Slowly activating voltage-gated potassium current potentiation by ML277 is a novel cardioprotective intervention
Source: PNAS Nexus. 2023 May 10;2(5):pgad156. doi: 10.1093/pnasnexus/pgad156 (PMC10208113; doi:10.1093/pnasnexus/pgad156)
Supplement: pgad156_Supplementary_Data [file pgad156_supplementary_data.docx]

**Supporting Information for**

**IKs potentiation by ML277 is a novel cardioprotective intervention**

Sean Brennan^1#^, Abrar IM Alnaimi^1#^, Lauren R McGuinness^1^, Muhammad IM Abdelaziz^1^, Robert A McKenzie^2^, Sophie Draycott^2^, Jacob Whitmore^1^, Parveen Sharma^1^, & Richard D Rainbow^1^*

Richard Rainbow

Email: [richard.rainbow@liverpool.ac.uk](mailto:richard.rainbow@liverpool.ac.uk)

**This PDF file includes:**

Supporting text for methods

Figures S1 to S8

Supporting Information Text

**Supplementary Methods**

**Isolation of ventricular myocytes.**

The care and sacrifice of the animals conformed to the requirements of the UK Animals (Scientific Procedures) Act 1986 (2012 amendment). Ethical approval for all experimental procedures was granted by the University of Leicester/Liverpool's Animal Welfare and Ethical Review Body (AWERB_2018_44). Adult male Wistar rats (200 – 300 g), or Dunkin-Hartley guinea pigs (up to 500 g), were killed by Schedule 1 procedure of concussion and cervical dislocation. Following Schedule 1, the heart was quickly removed from the thoracic cavity and briefly submerged into cold (4°C) Ca^2+^-free Tyrode's solution containing (in mM): KCl 5, NaCl 135, NaH_2_PO_4_ 0.33, Na pyruvate 5, HEPES 10, Mannitol 15, Glucose 5, MgCl 1, EGTA 0.3, to halt contractions and to lower the metabolic demand. The isolated heart was then cannulated via the aorta and mounted on a Langendorff type apparatus and perfused in a retrograde manner with warmed Ca^2+^-free Tyrode's solution (37°C) for 6 min. The enzyme mix solution (19 mg collagenase; 50 mg BSA prepared from factor V albumin and 16 mg protease; type XIV 15% Ca^2+^, Sigma-Aldrich) was then perfused through the heart until isolated cells appeared in the perfusate sampled from the ventricles. The solution was then exchanged for Ca^2+^-free Tyrode's solution for a further 2 min and then the heart cut down, washed in normal Tyrode’s (NT) solution containing (in mM): KCl 5, NaCl 135, NaH_2_PO_4_ 0.33, Na pyruvate 5, HEPES 10, Mannitol 15, Glucose 5, MgCl 1, CaCl_2_ 2, and cardiomyocytes mechanically separated using a shaking water bath at 37°C. This technique yielded 70–90% rod-shaped cardiomyocytes, which were stored in NT solution at room temperature and used within 12 h of isolation.

**HEK293 culture and transfection:**

Human embryonic kidney 293 (HEK293) cells were transiently transfected using FuGENE®6 with KCNQ1 and KCNE1 cDNA in a 2:1 ratio. Both *KCNQ1* and *KCNE1* genes were cloned into a pIRES2-EGFP vector and so transfected cells were identified by their green fluorescence under 480 nm epifluorescent illumination. HEK293 cells were maintained in Dulbecco’s Modified Eagle Medium (DMEM) containing 10% serum FBS and 1% penicillin / streptomycin.

HEK293 cells stably expressing hERG subunits were maintained in Dulbecco’s Modified Eagle Medium (DMEM) containing 10% serum FBS (myoclone) and the selection antibiotics gentamycin and geneticin (G418).

**Patch-clamp electrophysiology.**

Patch electrodes were made from filamented thick-walled borosilicate glass with a resistance of 3-6 MΩ. Recordings were made from isolated cardiomyocytes using an Axopatch 200B amplifier, filtering at 2 kHz. Recordings were digitized using a Digidata 1440 and recorded and analyzed using pCLAMP 10.3 software (Axon Instruments, Scientifica, Uckfield UK).

**Whole-cell:** Intracellular electrode solution contained (in mM) 30 KOH, 110 KCl, 10 EGTA, 10 HEPES, 1 MgCl_2_, 1 Mg-ATP, 0.1 Na-ADP, 0.1 GTP plus 20 nM CaCl_2_, pH 7.2 with HCl.

**Action potential (AP):** In current-clamp mode, APs were stimulated at 1 Hz via the patch electrode with a 5-ms depolarizing trigger, set to 130% of that required to elicit an AP (approximately 500-900 pA). Action potential duration to 90% repolarized (APD_90_) and membrane potential (Vm) were calculated within pCLAMP software offline (1-3).

**Whole cell currents:** In voltage-clamp mode, various protocols were used, these are shown in relevant figures in the results section (1-3). In all cases, one recording was made per cell.

**Simulated ischemia and reperfusion model.**

Freshly isolated ventricular cardiomyocytes were stimulated to contract using electric field stimulation (EFS) at 1 Hz. Contractile function was observed via a JVC CCTV camera and recorded to DVD for offline analysis. For simulated ischemia and reperfusion (I/R) experiments, cardiomyocytes were perfused (5 ml/min at 32-34°C) with NT solution for 2 min and substrate-free metabolic inhibition Tyrode's solution (SFT-MI; containing 2 mM cyanide and 1 mM iodoacetic acid) for 7 min, followed by 10 min of NT (reperfusion). Analysis was performed on the video file recordings to measure contractile and morphological changes of cardiomyocytes, such as contractile recovery after simulated ischemia, time to contractile failure and cell death (1-3).

**Video-edge detection:**

Cardiomyocytes were simulated to contract using 1 Hz EFS and contractile responses in the presence and absence of ML277 were recorded as outlined above. DVD recordings were played back and video-edge detection measurements were made using JVC CCTV camera connected through a Video-edge detection system (VED-105, Crescent electronics)/ The output was digitised using a Minidigi1B (Molecular Devices) and recorded to Axoscope 10.7 software (Molecular Devices). Area under the curve analysis was carried out using Graphpad Prism 9 on sequential contractions in the absence of, and following 5 minute of perfusion with, ML277. Mean data from 10 sequential contractions was used to create a single data point for each of the parameters shown (duration of contraction, amplitude of contraction, area under the curve, and the rates from baseline to peak and from peak back to baseline).

**Fluorescence imaging:**

To record calcium transients, cardiomyocytes were loaded with 5 μM Fura-2-AM for 20 minutes at room temperature. Cells were stimulated to contract at 1 Hz using electric field stimulation (EFS) and perfused at 32°C. Data was acquired using Winfluor4.2 software (Strathclyde University), with 340 and 380 nm excitation illumination provided by a PTI DeltaRam X monochromator. Emissions were collected using Andor Zyla4.5 camera at wavelength greater than 520 nm. Images were acquired at a rate of 26 ratios per second.

For more detailed analysis of the Ca^2+^ transients, cells were loaded with 5 μM Fluo-4-AM for 20 minutes. Cells were stimulated at 1 Hz using EFS and perfused with NT solution in the absence and presence of ML277 for 5 minutes each. Transients were recorded for 1 min in each condition, to limit photobleaching, at a rate of 40 images per second. Area under the curve analysis was carried out using Graphpad Prism 9 on sequential transients in the absence of, and following 5 minute of perfusion with, ML277. Mean data from 10 sequential transients was used to create a single data point for each of the parameters shown (duration of transient, amplitude of transient, area under the curve, and the rates from baseline to peak and from peak back to baseline).

For simultaneous measurements of calcium and mitochondrial membrane potential, cells were loaded with both 5 μM Fluo-4-AM and 1 μM Tetramethylrhodamine methyl ester (TMRM). At this concentration, TMRM accumulates in the mitochondria and auto quenches the signal. On depolarization of the mitochondria, the TMRM dissipates into the cytoplasm and the fluorescence increases. Fluorescence was excited alternately at 480 and 540 nm to excite the Fluo-4 and TMRM once every 5 seconds. Cells were perfused at 32 °C with normal Tyrode’s solution for 3 min followed by the metabolic inhibition Tyrode’s solution for 7 min. Cells were quiescent throughout to reduce movement artefacts, given that these were single wavelength indicators.

**Langendorff-perfused heart and coronary artery ligation.**

Following Schedule 1 killing by concussion and cervical dislocation, hearts from adult male Wistar rats (300–400 g) were quickly removed and cannulated on a Langendorff apparatus and perfused retrogradely with warmed Ca^2+^-free Tyrode's solution (37°C) for 1 hr to stabilize. The left anterior descending coronary artery was then ligated for 40 minutes to cause ischemia, by using 5-0 USP braided silk suture and two pipette tips to form a reversible knot around the artery (1). The knot was then removed to start the 3 hour reperfusion phase with the suture remaining in place to allow for re-ligation. During all phases temperature was carefully maintained at 37°C by submerging the heart in NT solution using a heated water jacket. Evans Blue dye (1% in NT solution) and 2,3,5-triphenyltetrazolium chloride (Sigma Aldrich) were used to identify area at risk (AAR) and infarcted area (IA), respectively. To determine the AAR and IA, each slice was scanned on both sides and weighed. AAR and IA were calculated for each of the slices from the heart using ImageJ. The AAR, IA and unaffected area sizes from ImageJ were then used to calculate the percentage infarct of the AAR by weight (1).

**Computer model of a human cardiac action potential:**

The human action potential was modeled using the O'Hara-Rudy CiPA v1.0 (2017) model running in OpenCOR software (4). For control conditions, the model was not modified from the original O'Hara-Rudy CiPA v1.0 (2017) model. Using data established in our HEK293 study (Supplementary figure 1) showing an ~30 mV leftward shift in activation curve and an approximate doubling of current, the model was modified to include a 30 mV leftward shift and a doubling of conductance to model the effect of ML277 on the human action potential, IKs current and intracellular Ca^2+^.

Equation 1: original steady-state activation gate (xs1ss) parameters where -11.6 mV is the mid-point of the activation curve with a slope factor of 8.932 mV

$$xs1ss=\frac{1}{1+e^{\frac{-(Vm+11.6)}{8.932}}}$$

Equation 2: Modified activation parameters to include 30 mV shift in the activation curve

$$xs1ss=\frac{1}{1+e^{\frac{-(Vm+11.6+30)}{8.932}}}$$

In the original model, xs2ss=xs1ss to represent the inactivation gate. Given that, equation one was used to represent xs2ss in the enhanced IKs model, given the assumption of no change in inactivation with ML277.

To simulate the steady-state, the model was run for 10 cycles and the data generated in the final cycle was used to plot the action potential, IKs current and intracellular calcium changes in Supplementary Figure 6.

**Supplementary data**


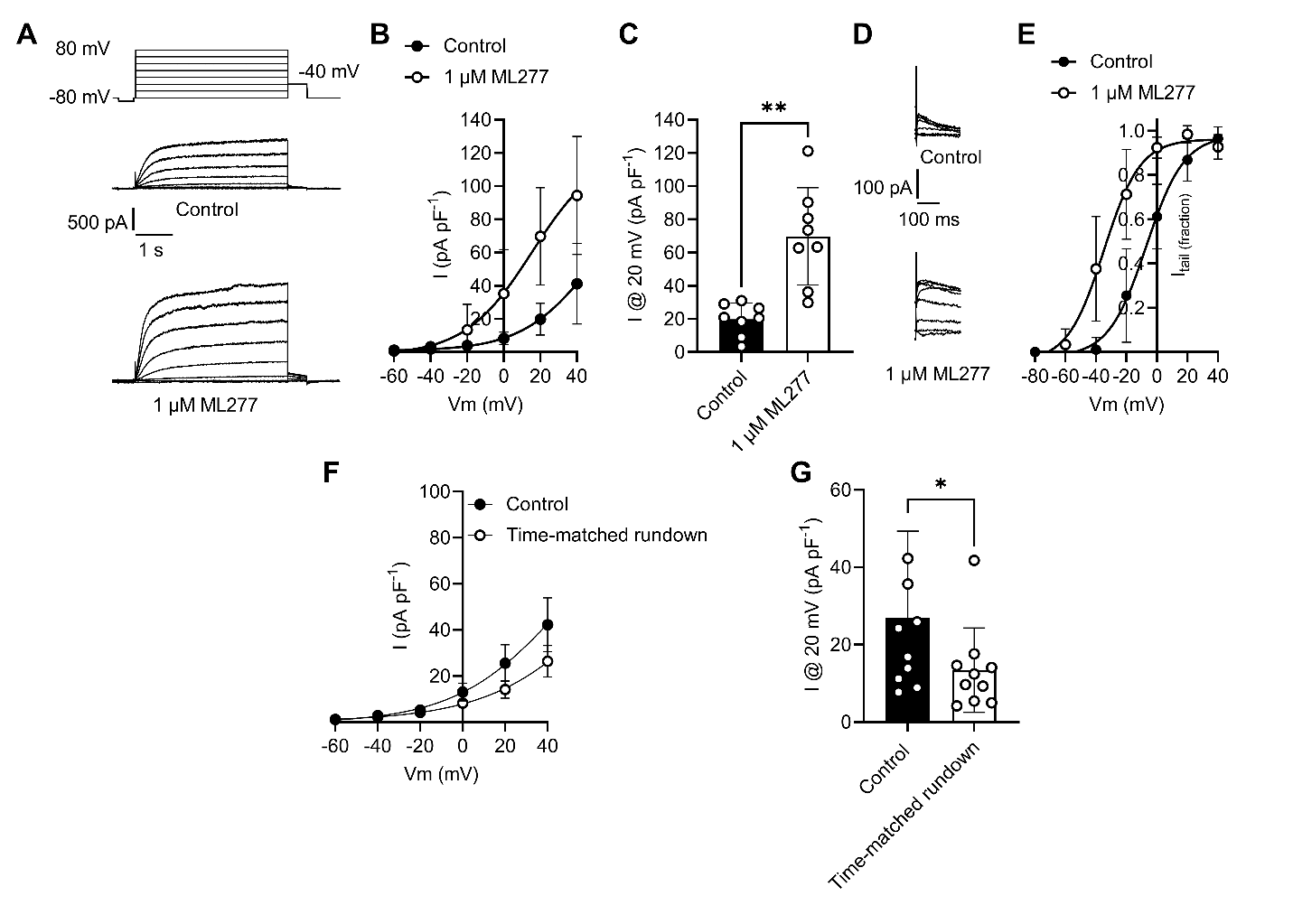


**Figure S1**

**ML277 potentiates KCNQ1/KCNE1 current transiently expressed in HEK293 cells.**

**(A)** Example current voltage relationship recordings from a HEK293 cell transiently transfected with KCNQ1/KCNE1 subunits in the absence and presence of 1 µM ML277. **(B)** Mean current-voltage relationship in the absence and presence of 1 µM ML277. **(C)** Mean whole-cell current density at 20 mV in the absence and presence of 1 µM ML277. (**P=0.0041, Paired t-test, n = 8). **(D)** Expanded tail currents from example traces in (A). **(E)** Mean activation curves plotted from the tail currents recorded at -40 mV with a half-maximal voltage of -6.9 ± 2.9 mV and -34.8 ± 3.4 mV in control and ML277 respectively. **(F)** Mean current-voltage relationship in control and time-matched rundown experiments in HEK293 cells **(G)** mean data at 20 mV showing the time matched rundown of KCNQ1/KCNE1 current (*P=0.0131, paired t-test, n = 9).


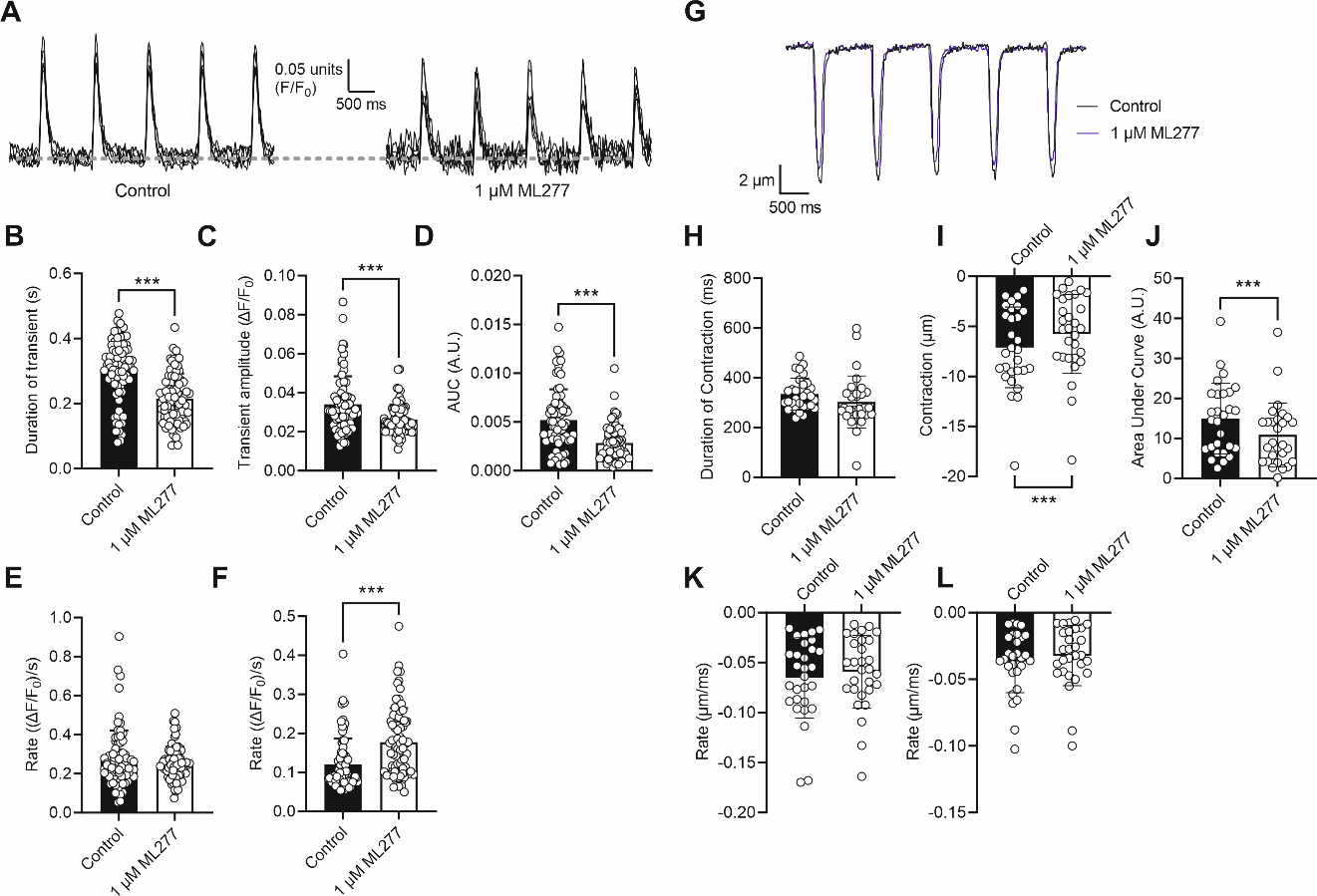


**Figure S2**

**ML277 significantly alters the calcium transients, but has limited effects on cardiomyocyte contraction.**

**(A)** Example calcium transients from 5 cells in the absence and presence of 1 µM ML277. **(B)** Mean duration, **(C)** mean amplitude, and **(D)** mean area under the curve for Ca^2+^ transients measured using Fluo-4, in 1 Hz electric field stimulation at 32 ± 2 °C, in the presence or absence of 1 µM ML277. **(E)** Mean rate from base to peak of the transient and **(F)** mean rate from peak to the return to the baseline. (***P<0.0001, Paired t-test, n = 75 cells from 5 experiments). **(G)** Example contractile responses from a single cardiomyocyte in the absence (black) and presence (blue) of 1 µM ML277. **(H)** Mean duration, **(I)** mean amplitude, and **(J)** mean area under the curve for contractions measured using video-edge detection, in 1 Hz electric field stimulation at 32 ± 2 °C, in the presence or absence of 1 µM ML277. **(K)** Mean rate from base to peak of the contraction and **(L)** mean rate from peak to the return to the baseline. (***P<0.0001, Paired t-test, n = 30 cells from 12 experiments).

*.*
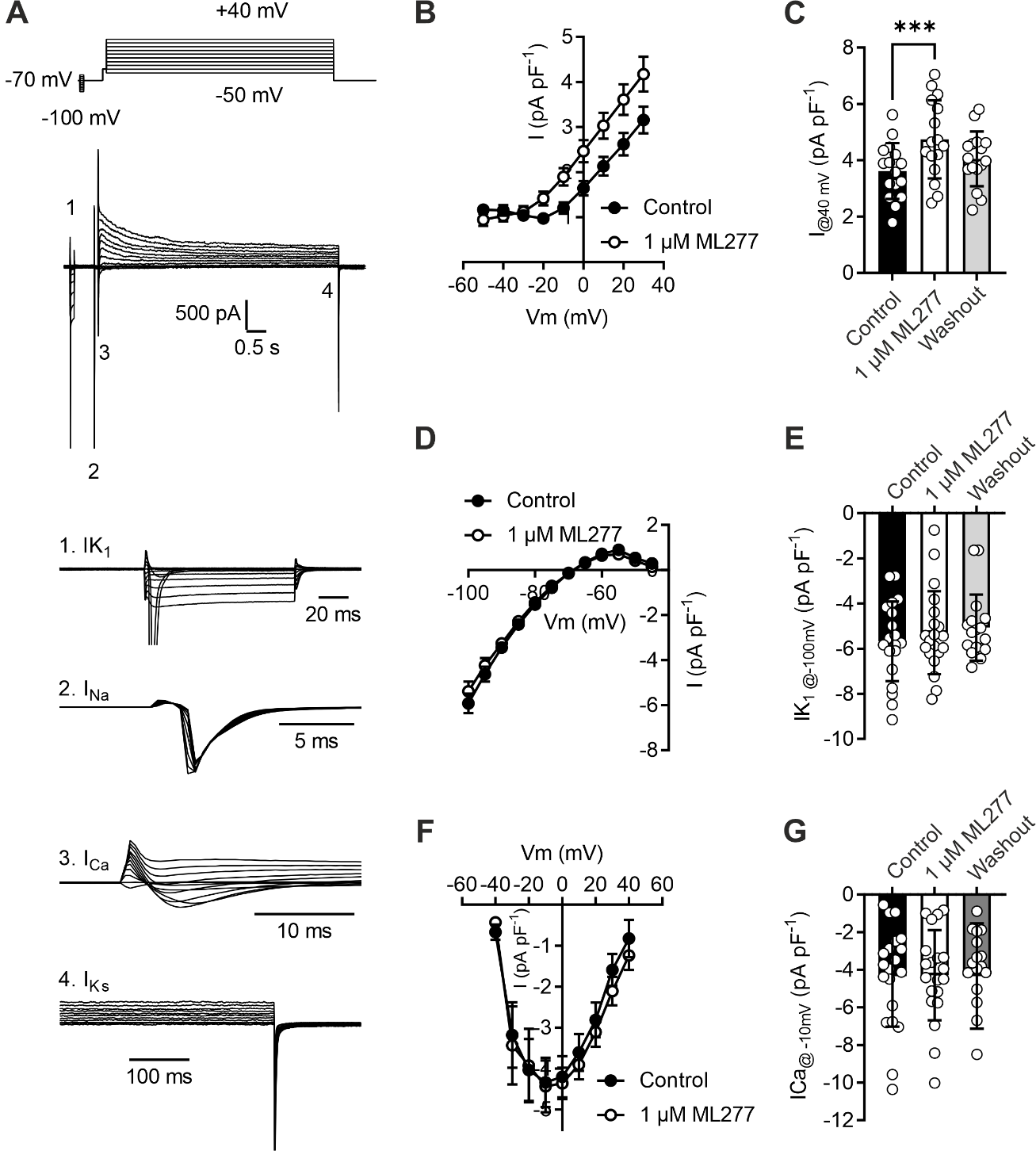


**Figure S3**

**ML277 potentiates delayed rectifier currents in rat ventricular cardiomyocytes but does not affect IK_1_ or calcium currents.**

**(A)** Example traces of the protocol and expanded traces showing **1)** inward rectifier current (IK_1_), **2)** Na currents (I_Na_), **3)** Calcium currents (I_Ca_) and **4)** Slowly activating K currents (I_Ks_), in control conditions. **(B)** Mean current voltage relationship showing before and after 5 min of perfusion with 1 µM ML277. **(C)** Mean data showing the mean delayed rectifier current at the end of a 6 s depolarization to 40 mV. There was a significant increase in delayed rectifier current that was lost following a 5 min washout of ML277 (**P<0.0001, Repeated-measures ANOVA with Dunnett’s Post-test, n = 16 cells). **(D)** Mean current-voltage relationship for I_K1_ current before and following 5 min perfusion with 1 µM ML277 **(E)** Mean current at -100 mV showing no significant difference between control, 1 µM ML277 or washout (Repeated-measures ANOVA, n = 16 cells). **(F)** Mean current-voltage relationship for I_Ca_ current before and following 5 min perfusion with 1 µM ML277 **(G)** Mean current at -10 mV showing no significant difference between control, 1 µM ML277 or washout (Repeated-measures ANOVA, n = 16 cells).


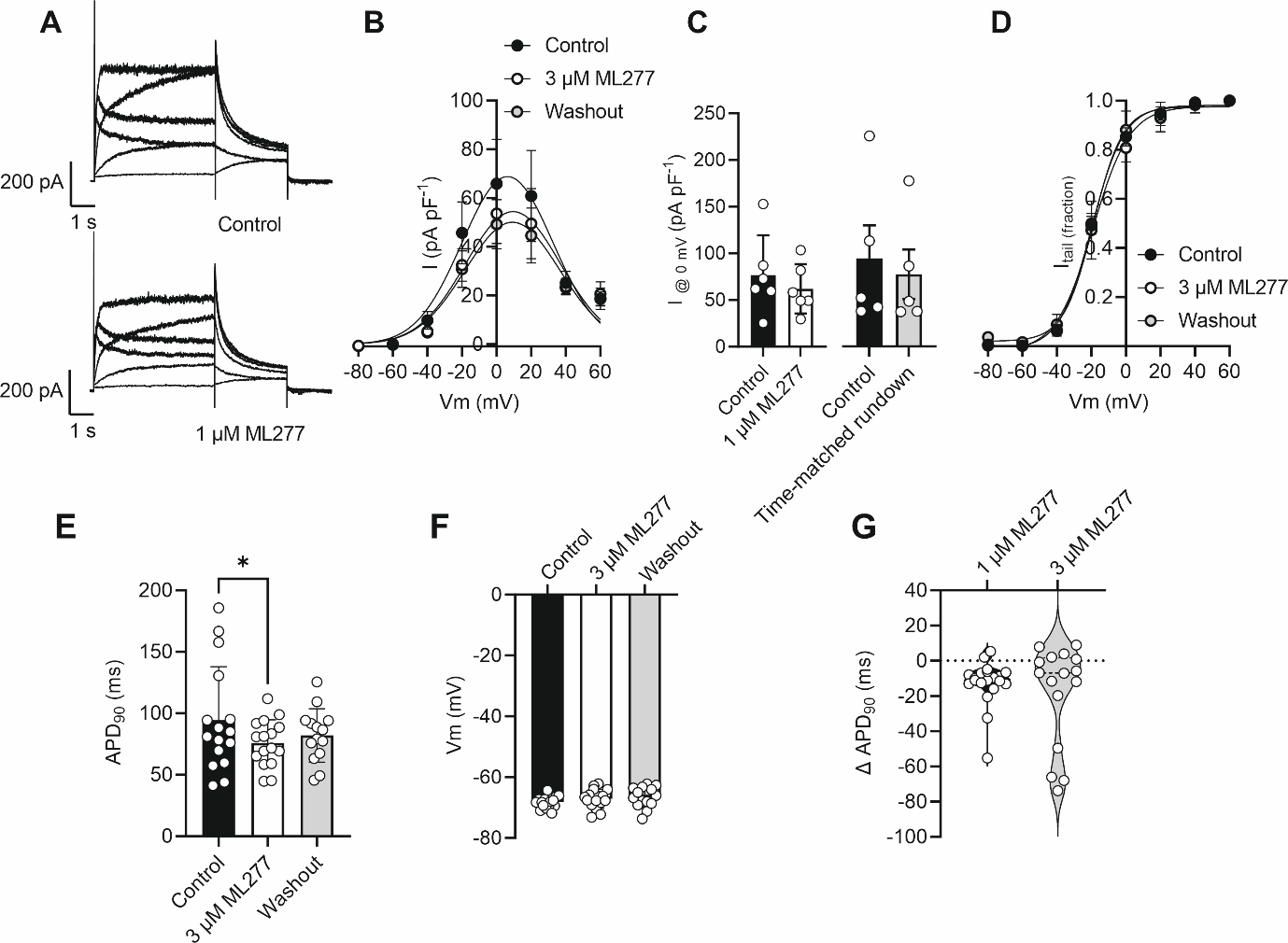


**Figure S4**

**A supramaximal concentration of ML277 (3 µM) had no effect on hERG current stably expressed in HEK293 cells or any further shortening of the cardiac action potential duration.**

**(A)** Example of a family of currents recorded from HEK293 cells stably expressing Kv11.1 and KCNE2 subunit. **(B)** Mean current voltage relationship from 6 cells showing control, 5 minutes of perfusion with 1 μM ML277 and following 5 minutes of washout. **(C)** Mean data from 1 μM ML277 treated, or time-matched rundown controls, showing no difference in the proportion of current rundown at 0 mV. **(D)** Mean tail current analysis showing no shift in the voltage-dependence of activation in control, 5 minute of perfusion with 1 μM ML277 and following 5 minute of washout. **(E)** Mean action potential duration in freshly isolated cardiomyocytes in control, 3 µM ML277 and washout solutions. **(F)** Mean membrane potential measurements for the same cells (*P=0.0370, Repeated Measures ANOVA with Dunnett’s post-test, n = 16 cells). **(G)** Comparison of the change in APD_90_ on perfusion with ML277 at 1 and 3 μM. There was no significant difference between the groups (P=0.3996, Unpaired t-test, n = 20 and 16, for 1 and 3 μM ML277 respectively.


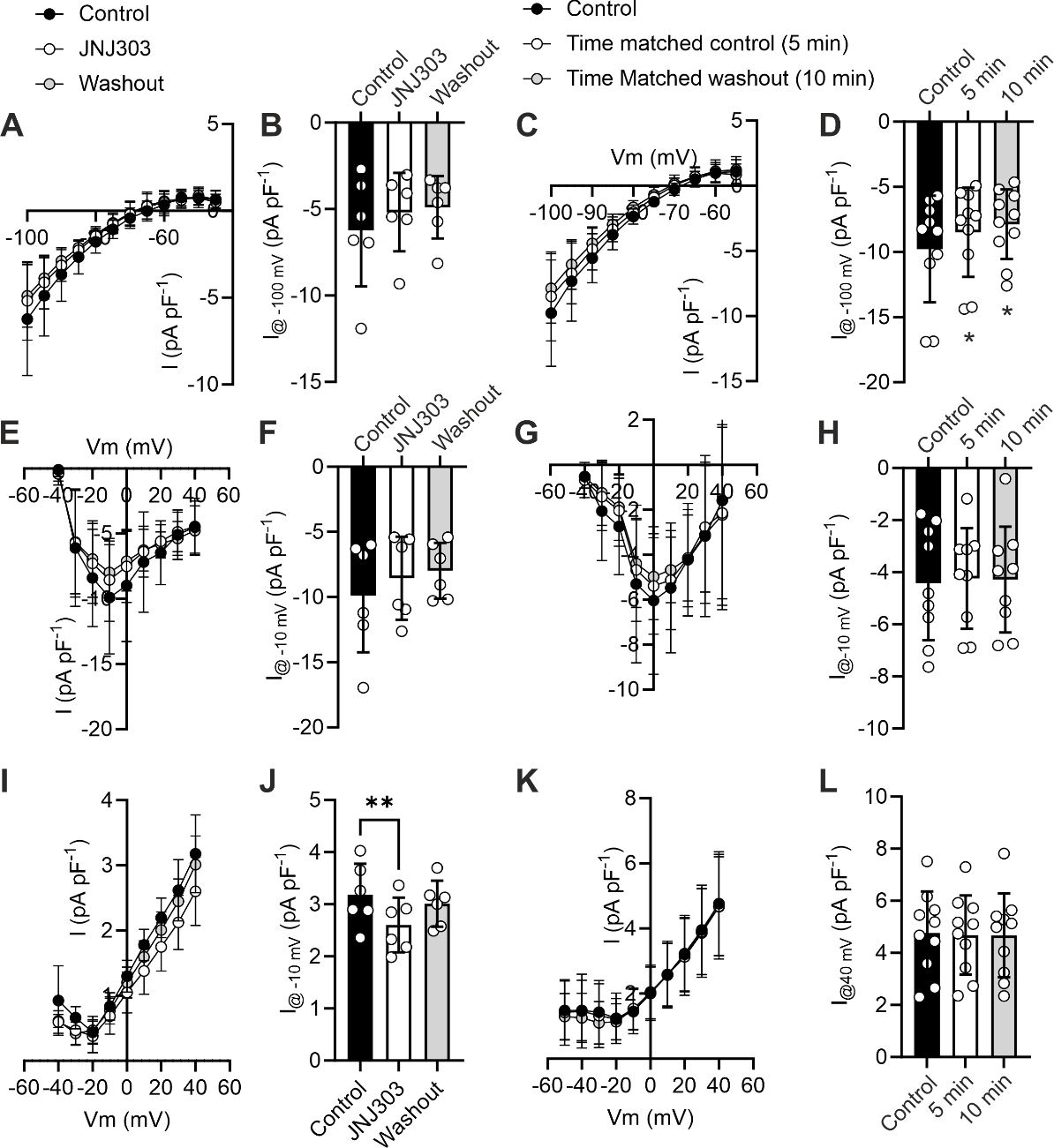


**Figure S5**

**1 μM JNJ303 inhibits delayed rectifier currents in rat ventricular myocytes but does not cause more inhibition of other currents than run down.**

All data recorded from an identical protocol as shown in Supplementary Figure 2 from rat isolated cardiomyocytes. **(A)** Mean current-voltage relationship for IK_1_ currents recorded in control conditions, with 1 µM JNJ303, and following 5 min washout. **(B)** mean current density at -100 mV in the conditions in (A). (Repeated-measures ANOVA, 6 cells). **(C)** Mean current-voltage relationship for IK_1_ currents recorded in control conditions, 5 min of perfusion as a time matched drug control and following 10 min of perfusion as a time-match for washout. **(D)** mean current density at -100 mV in the conditions in (C) (*P=0.045 and 0.041 for 5 and 10 min respectively, Repeated-measures ANOVA, 10 cells). **(E)** Mean current-voltage relationship for calcium currents in the conditions outlined in (A). **(F)** mean current density at -10 mV as in the conditions outlined in (A). (Repeated-measures ANOVA with Dunnett’s, 6 cells). **(G)** Mean current-voltage relationship for calcium currents in the conditions outlined in (C). **(H)** mean current density at -100 mV in the conditions in (C) (Repeated-measures ANOVA, 10 cells). **(I)** Mean current-voltage relationship for delayed rectifier currents in the conditions outlined in (A). **(J)** mean current density at 40 mV in the conditions outlined in (A). (**P=0.0064, Repeated-measures ANOVA with Dunnett’s post-test, 6 cells). **(K)** Mean current-voltage relationship for delayed rectifier currents in the conditions outlined in (C). **(L)** mean current density at 40 mV in the conditions outlined in (C). (Repeated-measures ANOVA, 10 cells).


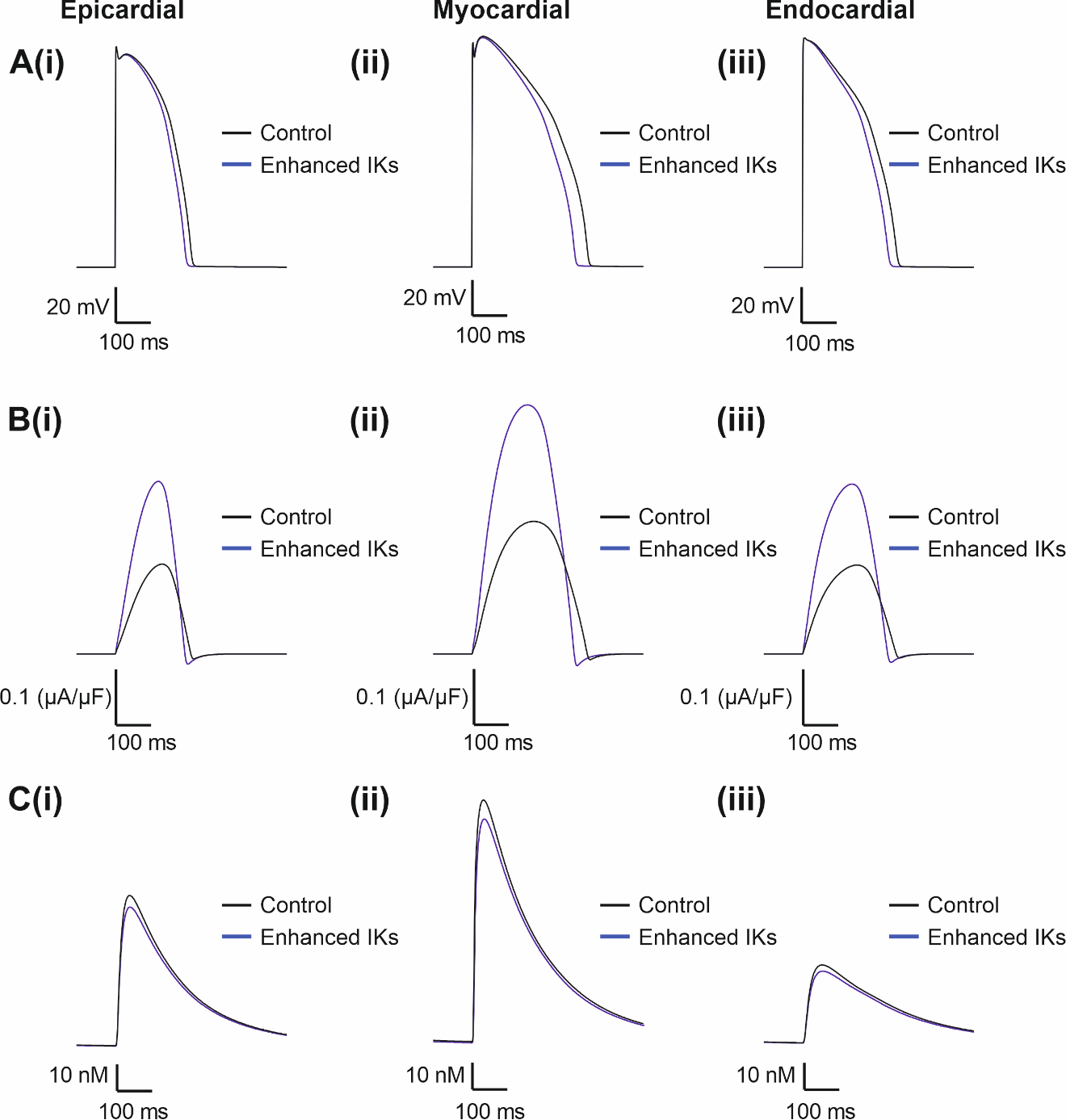


**Figure S6**

**Computer simulation of the effects of enhanced IKs activity on the human cardiac action potential.**

Responses in control conditions, and with IKs enhancement as seen using ML277, were modeled using the O'Hara-Rudy CiPA v1.0 (2017) model running in OpenCor software. Figures showing simulations of cardiac action potential **(A),** IKs current **(B)** and intracellular calcium change **(C)** in Epicardial (i), myocardial (ii) and endocardial myocytes in control conditions (unaltered model) and with enhanced IKs current. The enhancements to IKs were simulated by modifying the equations from the computer model to match the parameters recorded in HEK293 cells transfected with KCNQ1/KCNE1, a 30 mV shift in activation curve and a doubling of current.


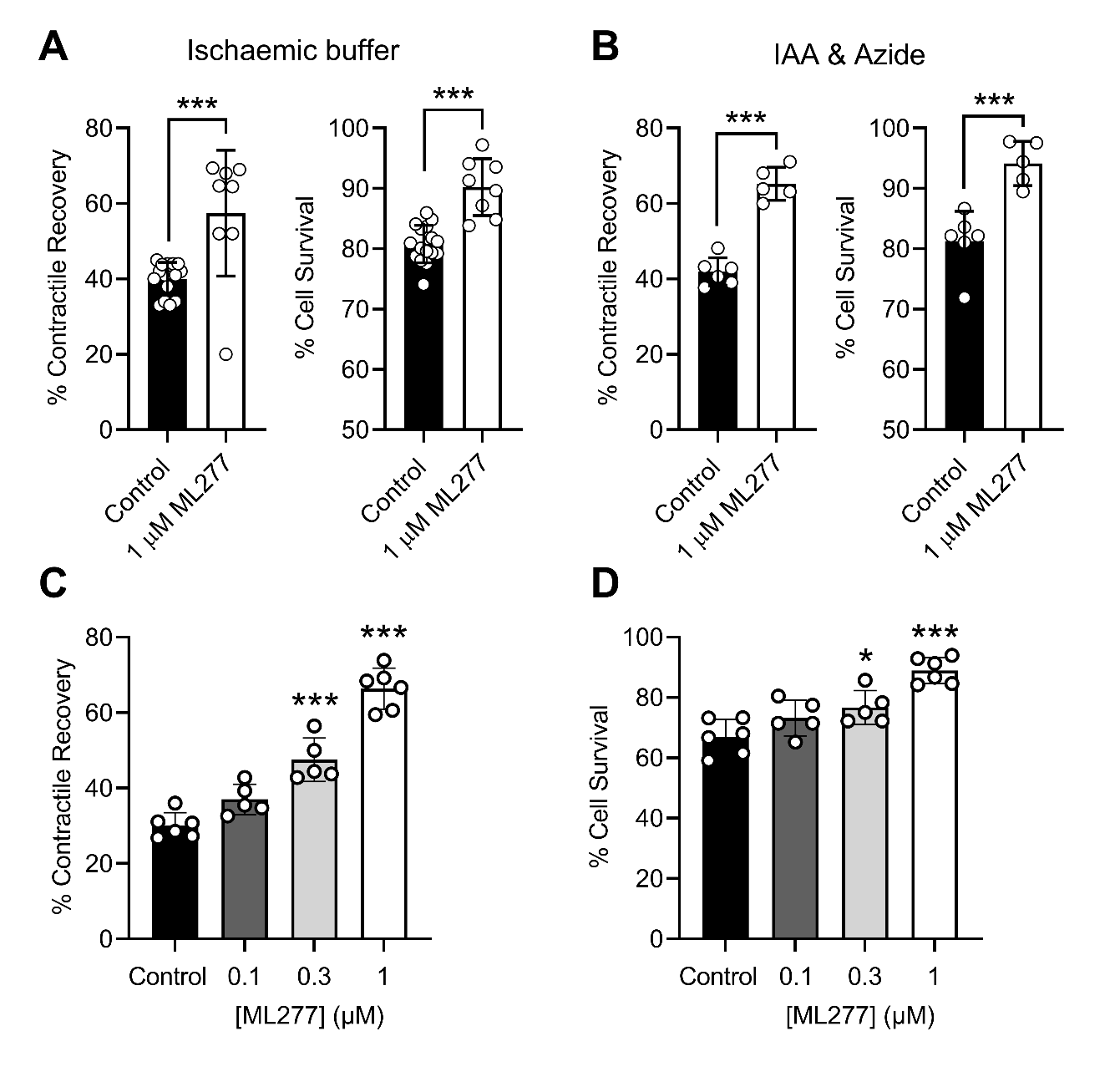


**Figure S7**

**ML277 is protective in rat cardiomyocytes from two additional metabolic inhibition protocols and protective in guinea pig isolated cardiomyocytes**

**(A)** Mean contractile recovery and cell survival data from an ischemic buffer protocol where the cells were treated with the ischemic buffer for 10 minutes, followed by 20 minutes washout. Similar to the data in figure 2, 1 μM ML277 imparted a significant cardioprotection, as evidenced by an increased contractile recovery and cell survival (***P=0.0006 and <0.0001, unpaired t-test, n = 16 (511 cells) and 8 (301 cells) control and ML277 respectively). **(B)** Mean contractile recovery and cell survival data from a substrate-free Tyrode’s solution containing 1 mM iodoacetic acid and 20 mM sodium azide. The metabolic inhibition was applied for 10 minutes followed by a 20 min washout. Again, 1 μM ML277 imparted a significant cardioprotection, as evidenced by an increased contractile recovery and cell survival (***P<0.0001 and P=0.001, unpaired t-test, n = 6 (224 cells) and 5 (196 cells) control and ML277 respectively). Guinea pig cardiomyocytes were exposed to the same protocol as outlined in Figure 2, demonstrating an ML277 concentration-dependent increase in **(C)** contractile recovery and **(D)** cell survival. (*P=0.0226, ***P<0.0001, One-Way ANOVA with Dunnett’s post-test, n = 6 (180), 5 (142), 5 (110), 6 (145) experiments (cells) for control, 0.1, 0.3 and 1 μM ML277 respectively).


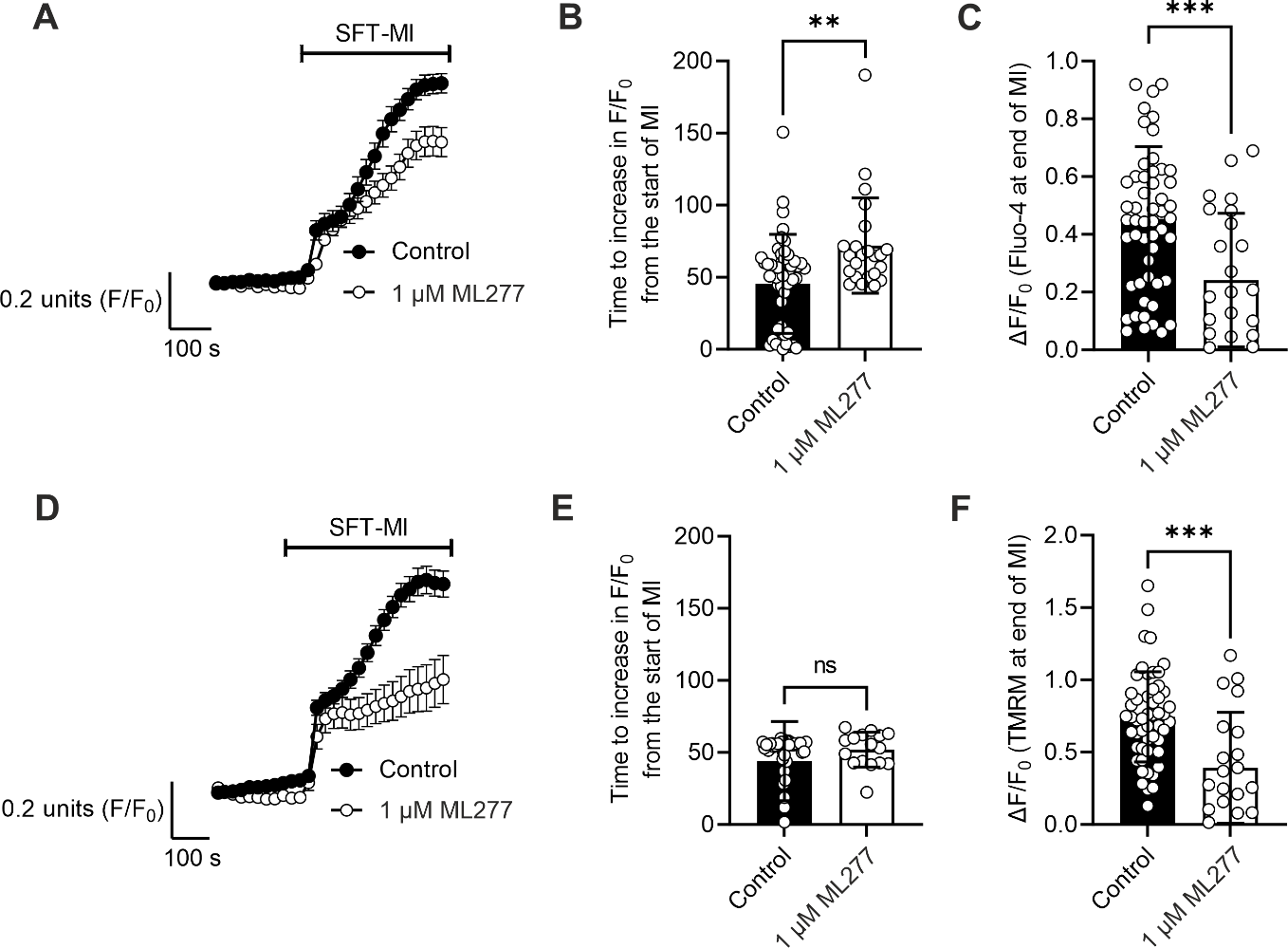


**Figure S8**

**Calcium accumulation and mitochondrial depolarization are altered with ML277 treatment in a metabolic inhibition and washout protocol.**

**(A)** Example time course data of the Fluo-4 signal from rat cardiomyocytes in the presence and absence of 1 μM ML277 in a metabolic inhibition and washout protocol. **(B)** Mean data showing the time to an increase of 10% above basal fluorescence in control and 1 μM ML277-treated cardiomyocytes (**P=0.0024, unpaired t-test, n = 53 control and 23 ML277 treated cells). **(C)** Mean data showing the change in Fluo-4 fluorescence from baseline at the end of the metabolic inhibition (***P=0.0007, unpaired t-test, n = 53 and 23, control and ML277 treated cells respectively). **(D)** Example time course data of the TMRM signal from the same rat cardiomyocytes as in (A) in the presence and absence of 1 μM ML277 in a metabolic inhibition and washout protocol. **(E)** Mean data showing the time to an increase of 10% above basal fluorescence in control and 1 μM ML277-treated cardiomyocytes (no significant difference unpaired t-test, n = 53 control and 23 ML277 treated cells). **(F)** Mean data showing the change in TMRM fluorescence from baseline at the end of the metabolic inhibition (***P=0.0001, unpaired t-test, n = 53 and 23 , control and ML277 treated cells respectively).

References:

1. S. Brennan *et al.*, A novel form of glycolytic metabolism-dependent cardioprotection revealed by PKCalpha and beta inhibition. *J Physiol* **597**, 4481-4501 (2019).

2. S. Brennan *et al.*, Early opening of sarcolemmal ATP-sensitive potassium channels is not a key step in PKC-mediated cardioprotection. *J Mol Cell Cardiol* **79**, 42-53 (2015).

3. M. W. Sims *et al.*, PKC-mediated toxicity of elevated glucose concentration on cardiomyocyte function. *Am J Physiol Heart Circ Physiol* **307**, H587-597 (2014).

4. S. Dutta *et al.*, Optimization of an In silico Cardiac Cell Model for Proarrhythmia Risk Assessment. *Front Physiol* **8**, 616 (2017).
